# De-Escalating Anti-Pseudomonal β-Lactams

**DOI:** 10.1186/2197-425X-3-S1-A3

**Published:** 2015-10-01

**Authors:** J Catteeuw, L De Bus, W Denys, B Gadeyne, JJ De Waele, J Decruyenaere, PO Depuydt

**Affiliations:** Ghent University Hospital, Department of Critical Care Medicine, Ghent, Belgium; Ghent University- iMinds, Department of Information Technology, Ghent, Belgium

## Introduction

De-escalation of broad-spectrum antibiotics such as anti-pseudomonal beta-lactams is recommended to reduce antimicrobial selection pressure.

## Objectives

To identify factors associated with de-escalation of anti-pseudomonal beta-lactams in the Intensive Care Unit (ICU). To assess whether de-escalation was associated with outcome as measured by ICU mortality and acquisition of MDR-pathogens.

## Methods

We retrospectively included all ICU episodes in Ghent University Hospital during 2013 and 2014 with prescription of meropenem, piperacillin-tazobactam or ceftazidime for at least 48 hours, stratifying between ICU episodes of < and ≥4 days; for outcome analysis, only the first ≥4 days episode per patient was included. De-escalation was defined as a change from meropenem to a non-carbapenem antibiotic other than colistin or aminoglycosides or a change from ceftazidime or piperacillin-tazobactam to a non anti-pseudomonal antibiotic. Organ failure improvement was defined as a decrease of the SOFA score between day 3 and day 1 of the infection for which the beta-lactam was prescribed. Antibiotics were considered appropriate if they covered all etiologic pathogens of the infection. Multidrug-resistant (MDR) pathogens were defined according to Magiorakos et al. (1). Acquisition of MDR pathogens was defined as the identification of MDR pathogens more than 2 days after the start of the antibiotic under study and not present before this date.

## Results

Anti-pseudomonal beta-lactam antibiotics (n = 609) were de-escalated in 23%: meropenem (n = 129) in 33% and piperacillin-tazobactam (n = 453) or ceftazidime (n = 27) in 20% (combined). De-escalation was not associated with the focus or severity of infection, but was significantly associated with organ failure improvement (p = 0.03) and with the identification of etiologic pathogens (p < 0.001). Total antibiotic duration in infections with and without de-escalation was 7 and 5 days, respectively (p < 0.001). in multivariable analysis examining the relationship between ICU-mortality and de-escalation with adjusment for organ failure improvement and focus and severity of infection, ICU mortality was not associated with de-escalation. Acquisition of MDR was not significantly different in episodes with or without de-escalation (33% versus 32%, p= 0.87).

## Conclusions

De-escalation was associated with availability of etiologic microbiological cultures and improvement of the SOFA score. De-escalation was not associated with ICU mortality or with the acquisition of MDR pathogens.

## Grant Acknowledgment

L. De Bus received a Clinical Research Grant from Ghent University Hospital, Belgium.
